# Astaxanthin Inhibits Ferroptosis of Hippocampal Neurons in Kainic Acid‐Induced Epileptic Mice by Activating the Nrf2/GPX4 Signaling Pathway

**DOI:** 10.1111/cns.70238

**Published:** 2025-02-17

**Authors:** Shihao Chen, Linqian Zhao, Xing Jin, Qichang Liu, Yuqing Xiao, Huiqin Xu

**Affiliations:** ^1^ Department of Neurology The First Affiliated Hospital of Wenzhou Medical University Wenzhou China; ^2^ Zhejiang Chinese Medical University Hangzhou China

**Keywords:** astaxanthin, epilepsy, ferroptosis, kainic acid, Nrf2/GPX4 pathway

## Abstract

**Background:**

Epilepsy, a prevalent neurological disorder, is distinguished by episodic abnormal discharges of neurons within the brain, resulting in transient brain dysfunction. Prior research has identified a novel form of cell death termed ferroptosis, which is intricately linked to the initiation and progression of epilepsy. It has been demonstrated that astaxanthin (AST) can inhibit ferroptosis by enhancing the activity of nuclear factor erythroid 2‐related factor 2 (Nrf2), thereby providing cytoprotection. Therefore, this study aims to investigate whether AST can alleviate neuronal ferroptosis in epilepsy by activating the Nrf2/GPX4 pathway, thereby exerting a neuroprotective effect.

**Methods:**

By constructing a kainic acid (KA)‐induced epilepsy mouse model and a KA‐induced HT22 cell model, we employed behavioral testing, Western blot analysis, quantitative real‐time reverse transcription qRT‐PCR, ferroptosis‐related assay kits, immunofluorescence staining, and other methods. These methodologies were utilized to investigate the protective effects and underlying mechanisms of AST on ferroptosis in KA‐induced epileptic mice and HT22 neurons.

**Results:**

Our results demonstrate that AST pretreatment alleviates KA‐induced epileptic behaviors and cognitive impairments in mice and mitigates ferroptosis indicators such as lipid peroxidation and mitochondrial morphological alterations. This neuroprotective effect appears to be mediated by the activation of the Nrf2/GPX4 signaling axis. In vitro studies further revealed that AST confers neuroprotection against KA‐induced HT22 neuronal cell death, an effect that is abrogated by an Nrf2 inhibitor. Hence, the neuroprotective properties of AST are significantly associated with the modulation of the Nrf2‐mediated ferroptosis pathway, as corroborated by bioinformatics analyses.

**Conclusion:**

The AST effectively inhibits neuronal ferroptosis in both in vivo and in vitro epilepsy models via the Nrf2/GPX4 pathway. This finding suggests that AST holds promise as a potential therapeutic agent for the treatment of epilepsy.

## Graphical abstract

The AST activates the Nrf2/GPX4 signaling pathway to regulate glutathione metabolism and inhibit ferroptosis to preventing epileptic seizures.

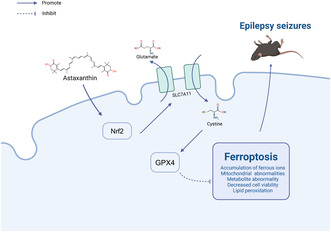

## 1 Introduction

Epilepsy, a prevalent neurological disorder, is characterized by sudden abnormal neuronal discharges in the brain, resulting in transient brain dysfunction that severely impacts patients' physical and mental health and daily life. According to the 2019 Global Burden of Disease (GBD) study, epilepsy affects over 50 million people worldwide. From 1990 to 2019 in China, the incidence of epilepsy increased by 45%, significantly exceeding the global rate [1]. Currently, epilepsy is the second most common neurological condition after migraines. Moreover, the primary demographic affected by epilepsy is predominantly middle‐aged and elderly men, where sudden seizures can result in severe injuries or accidental death [2]. Hence, early detection and treatment are particularly crucial, and understanding the pathogenesis of epilepsy and discovering suitable treatments will be beneficial.

The pathogenesis of epilepsy is multifaceted, encompassing genetic factors and triggers such as brain and systemic diseases [3, 4]. It is widely accepted that epilepsy involves an imbalance between excitation and inhibition in the central nervous system, potentially related to ion channels, neuronal loss, and glial cell changes [5, 6, 7, 8]. Previous studies have indicated that a novel form of cell death, ferroptosis, is associated with the onset and progression of epilepsy [9].

Ferroptosis was first introduced by Dr. Brent R. Stockwell of Columbia University in 2012 [10]. It involves the iron‐mediated accumulation of lipid peroxides, where an excess of polyunsaturated fatty acids integrates into the cell membrane, causing membrane rupture and cell death [11]. Ferroptosis is closely linked to neurological diseases. Research has shown that in Parkinson's disease, elevated cellular iron content accelerates the formation of α‐synuclein fibrils, contributing to the disease [12, 13]. In Alzheimer's disease, knocking out the GPX4 protein in mouse brains results in typical symptoms of neurological dysfunction and cognitive impairment, which can be somewhat alleviated by ferroptosis inhibitors [14]. Regarding epilepsy, dysregulation of iron metabolism has been reported in temporal lobe epilepsy [15], and certain ferroptosis‐targeting methods have been reported to reduce neuronal damage in epilepsy [16]. Thus, ferroptosis may play a role in the progression of neurological diseases, and inhibiting ferroptosis could provide novel insights for treatment.

Nuclear factor erythroid 2‐related factor 2 (Nrf2) is a key factor in ferroptosis, acting as a transcription factor that regulates the expression of antioxidant response elements. Kelch‐like ECH‐associated protein 1 (Keap1) is an endogenous inhibitor of Nrf2. Under oxidative stress conditions, Keap1 degrades and dissociates from Nrf2, allowing Nrf2 to translocate to the nucleus and initiate the transcription of genes harboring antioxidant response elements [17], thereby promoting cellular antioxidant activity through increased expression of target genes like glutathione peroxidase 4 (GPX4) [18]. GPX4 is a protein that protects cell membranes from lipid peroxidation and converts reduced glutathione (GSH) to oxidized glutathione (GSSG) [19]. Inhibition of GPX4 leads to ferroptosis through the accumulation of lipid peroxides. A recent study has identified Nrf2‐mediated ferroptosis in epilepsy models [20].

Astaxanthin (AST), an orally administered carotenoid, possesses strong antioxidant and anti‐inflammatory properties, aiding in protecting cells from free radical damage and slowing the aging process [21, 22]. Its ability to effectively cross the blood–brain barrier suggests that it may help prevent or reduce the severity of certain neurological diseases, such as Parkinson's [23], Alzheimer's [24] and multiple sclerosis [25]. It has been demonstrated that AST can inhibit oxidative damage and inflammation by enhancing the activity of Nrf2 and the expression of its downstream antioxidant enzymes, thereby providing cytoprotection [26, 27]. Nevertheless, whether AST controls seizures via the ferroptosis mechanism remains unexplored. Thus, we aim to investigate the pathogenesis and development of epilepsy from the perspective of ferroptosis, as well as the neuroprotective effects of AST.

In summary, this study aims to explore the neuroprotective mechanism of AST against KA‐induced epilepsy in mice by examining the ferroptosis through the Nrf2/GPX4 signaling pathway.

## 2 Materials and Methods

**FIGURE 1:**
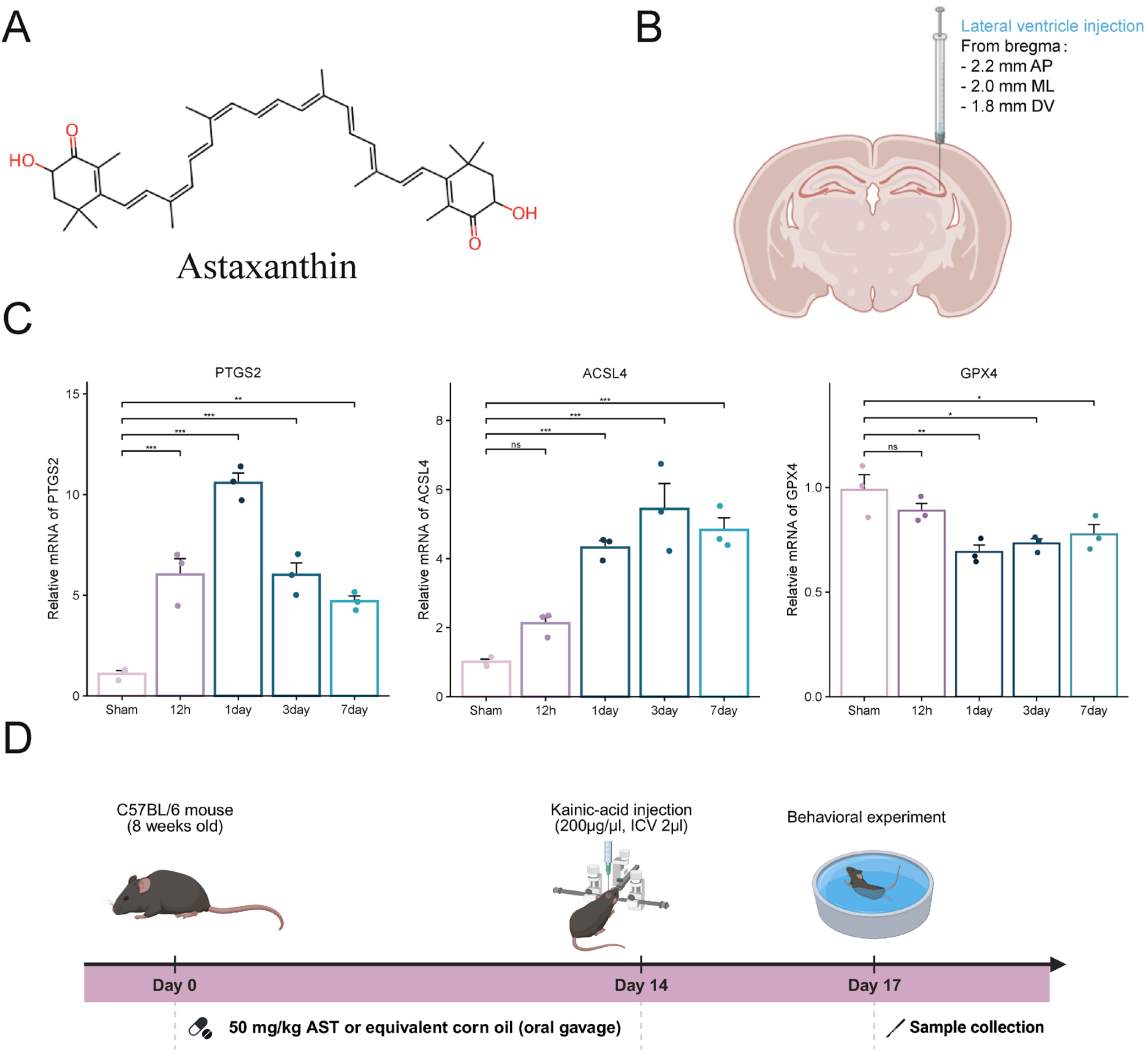
(A) The chemical structure of AST. (B) Stereotactic modeling site in KA‐induced epileptic mice. (C) Expression levels of ACSL4, PTGS2, and GPX4 mRNA in the hippocampal tissues of KA‐induced epileptic mice (*n* = 3). Data are presented as mean ± SD. *p < 0.05, **p < 0.01, ***p < 0.001. (D) Experimental design workflow.

Adult C57BL/6 mice (8 weeks old, weighing 20–25 g) were procured from the Experimental Animal Center of Wenzhou Medical University. The mice were housed under controlled laboratory conditions, maintaining a 12‐h light/dark cycle and a temperature of 24°C ± 2°C. They had unrestricted access to food and water. The mice were randomly allocated to three groups: sham, KA, and KA + AST (treatment) groups. AST (≥ 98% purity, HPLC, mixture of cis and trans isomers) was purchased from Aladdin, with its chemical structure illustrated in Figure 1A. Based on prior studies [23, 28], AST was dissolved in corn oil to achieve a concentration of 50 mg/kg and administered via oral gavage to the KA + AST group. The KA and sham groups received an equivalent volume of corn oil by gavage. After a 14‐day gavage period, mice were intraperitoneally injected with pentobarbital sodium (50 mg/kg) and secured in a stereotaxic apparatus. According to hippocampal coordinates determined by our research team (AP −2.2 mm, ML −2.0 mm, V −1.8 mm), as depicted in Figure 1B, KA obtained from MCE was diluted to 200 ng/μL and administered into the hippocampus at a rate of 0.4 μL/min for a total volume of 2 μL. Post‐injection, the needle was maintained in position for 5 min to prevent backflow. The sham group received an equal volume of saline at the same coordinates. Seizure severity was assessed 2 h post‐surgery using the Racine scale, which classifies seizures as follows: Stage 0: No response. Stage 1: Facial clonus, including clonic twitches of facial muscles. Stage 2: Stage 1 symptoms combined with rhythmic head nodding. Stage 3: Stage 2 symptoms along with clonic twitches of the forelimbs. Stage 4: Stage 3 symptoms coupled with rearing, typically accompanied by clonic twitches of the forelimbs and stiffening of the hind limbs. Stage 5: Stage 4 symptoms combined with falling and generalized clonic seizures. Only mice with Racine scores of 4–5 were included in the subsequent study. All experimental procedures were reviewed and approved by the Ethics Committee of Wenzhou Medical University and conducted in accordance with the National Institutes of Health Guide for the Care and Use of Laboratory Animals.

### 2.1 RT‐qPCR

Hippocampal samples were collected for RT‐qPCR analysis. Total RNA was extracted using Trizol reagent (Takara Bio, Tokyo, Japan), and cDNA was synthesized from 1 μg of RNA using the PrimeScript RT reagent kit (Takara Bio, Tokyo, Japan). RT‐qPCR analysis was performed using SYBR Green PCR Master Mix (Takara Bio, Tokyo, Japan) and appropriate primers on an Opticon 2 Real‐Time PCR Detection System (Bio‐Rad). PCR amplification was conducted using a protocol consisting of an initial step at 95°C for 5 min, followed by 40 cycles of 95°C for 1 min, 62°C for 30 s, and 72°C for 30 s. The mRNA expression levels of target genes were normalized to the housekeeping gene β‐actin and calculated using the 2^−ΔΔ*Ct*^ method. Results are expressed as the mean ± SEM of triplicate samples from three independent experiments.

### 2.2 Cell Experiments

To investigate the effect of AST on KA‐induced neuronal death, we used a KA‐induced neuron death model as an in vitro model of epilepsy [29]. Briefly, immortalized hippocampal neurons were cultured in DMEM (D5796, Sigma–Aldrich, St. Louis, USA) supplemented with 10% fetal bovine serum, 100 units of penicillin, and 100 μg/mL streptomycin. Cells were seeded in 6‐well or 96‐well plates and incubated at 37°C in a 5% CO_2_ atmosphere for 24 h before the experiment. Experiments were conducted according to the grouping of the animal experiments. Regarding the administration of AST to HT22 cells, we referred to the study by Wen et al. [30], when the cell density reaches 70%–80%, pre‐treat the cells with AST for 2 h, followed by incubation with KA for 12 h to evaluate the protective effect of AST.

To determine the suitable concentration of AST and KA for modeling, we used the Cell Counting Kit‐8 (C0038, Beyotime, Shanghai, China) to measure cell viability. In brief, cells were seeded in 96‐well plates at a density of 5 × 10^3^ cells/well in a volume of 100 μL/well. After treatment, 10 μL of CCK8 solution was added to each well and incubated for 30 min. Cell viability was then measured at 450 nm using a microplate reader.

### 2.3 Western Blot

Western blot analysis was used to quantify protein level changes in hippocampal or HT22 cells. Briefly, samples were boiled at 100°C for 10 min, separated by 10% sodium dodecyl sulfate‐polyacrylamide gel electrophoresis (SDS‐PAGE), and then transferred to PVDF membranes (Millipore, Billerica, MA, USA). Membranes were blocked with 5% nonfat milk in Tris‐buffered saline with 0.1% Tween (TBST) for 1.5 h at room temperature, and then incubated overnight at 4°C with primary antibodies at appropriate dilutions. After three washes with TBST, membranes were incubated with HRP‐conjugated secondary antibodies at room temperature for 1 h. The antibodies used for Western blot analysis are detailed in Table S1. Detection was performed using an ECL‐Enhanced Chemiluminescent Reagent Kit (NCM Biotech, USA). Band densities were quantified using Image J software (Pierce, Rockford, USA) and normalized to β‐actin.

### 2.4 Transmission Electron Microscopy

Mitochondrial morphological changes are considered a key phenotypic characteristic of ferroptosis. To investigate this phenomenon in detail, we performed transmission electron microscopy (TEM) analysis on hippocampal tissue. Briefly, fresh tissue was fixed in 2.5% glutaraldehyde in phosphate‐buffered saline (PBS) for over 4 h, followed by fixation in 1% OsO4 in PBS for over 2 h. The samples were then dehydrated in a graded series of ethanol and acetone, and embedded in Epon 816 (Electron Microscopy Sciences, Hatfield, PA, USA). Ultrathin sections were prepared using an RMC Boeckeler PowerTome XL ultramicrotome and stained with uranyl acetate and lead citrate for 5 and 10 min, respectively. TEM images were captured using a Hitachi H‐7550 transmission electron microscope (Hitachi, Tokyo, Japan).

### 2.5 Measurement of Lipid Peroxidation and Ferrous Ion Levels

Lipid peroxidation and ferrous ion accumulation are additional key phenotypic features of ferroptosis. To quantify lipid peroxidation, glutathione, and ferrous ion levels in hippocampal tissue and HT22 cells, we used commercial kits as described in previous literature [20]. Malondialdehyde (MDA) levels were measured using an MDA Assay Kit (S0051, Beyotime, Shanghai, China), glutathione (GSH) levels using a GSH Assay Kit (S0053, Beyotime, Shanghai, China), and ferrous ion levels using a Ferrous Ion Content Assay Kit (BC5415, Solarbio, Beijing, China). In summary, after preparing hippocampal or cell homogenates, thiobarbituric acid (TBA), glutathione reductase, and 2,4,6‐tri(2‐pyridyl)‐1,3,5‐triazine (TPTZ) were added separately. Under elevated temperatures and acidic conditions, MDA reacts with TBA to form a red MDA–TBA complex. Glutathione reductase facilitates the reduction of oxidized glutathione to its reduced form, GSH, which subsequently reacts with the chromogenic substrate DTNB, resulting in a yellow TNB product. Additionally, ferrous ions form a blue complex with TPTZ in acidic conditions. Once the reactions are complete, measure the absorbance at 532 nm (MDA), 412 nm (GSH), and 593 nm (ferrous ions) using a microplate reader. Additionally, HT22 cell ferrous ion levels were assessed using the Cell Ferrous Iron Fluorometric Assay Kit (MA0647, MeilunBio, China). After removing the culture medium, cells were washed 2–3 times with cell staining buffer and incubated with staining working solution for 60 min at 37°C in a 5% CO_2_ incubator. Without washing, the cells were observed using a Nikon confocal microscope.

### 2.6 Nissl Staining

To explore the potential neuroprotective effects of AST, we conducted Nissl staining. Mice were anesthetized and perfused transcardially with PBS, followed by 4% paraformaldehyde. The brains were extracted, dehydrated, and paraffin‐embedded. Paraffin sections were then subjected to Nissl staining. Brain sections (5 μm) were incubated with Nissl staining solution (C0117, Beyotime, Shanghai, China) for 15 min. Standard processing was performed, and images were captured using a Nikon optical microscope (Nikon, Tokyo, Japan).

### 2.7 Behavioral Experiments

To investigate the potential impact of AST on cognitive function impairment 3 days post‐KA injection, we conducted Morris water maze (MWM) and novel object recognition tests as previously reported [20]. In brief, the MWM was conducted in a white pool with a diameter of 120 cm. During the navigation test, mice underwent training for 4 consecutive days, with three trials per day. In each trial, mice were required to locate a hidden platform submerged 1 cm below the water surface within 60 s. On the fifth day, a probe test was conducted by removing the platform and allowing the mice to search for it for 60 s from the quadrant opposite the previous platform location. The number of times the mice crossed the platform area and their movement tracks were recorded using SMART 3.0 software.

Additionally, we conducted a novel object recognition test in an arena measuring 45 cm × 45 cm × 30 cm. Mice were initially acclimatized to the empty arena for 5 min. During the training phase, mice explored two identical square objects for 20 min. A memory test was conducted 24 h later, during which one square object was replaced with a triangular object, and exploration was recorded for 5 min. The time spent exploring the new object (ET) and the old object (ES) was recorded. The recognition index (RI) was calculated as RI = ET/(ES + ET), with RI > 0.5 indicating a preference for the new object. Movement tracks and exploration times were tracked and recorded using SMART 3.0 software equipped with a video tracking system.

### 2.8 Immunofluorescence

Mice were perfused transcardially with PBS followed by 4% paraformaldehyde. The brains were then immersed in 30% sucrose for 3 days, and sections (25 μm) were prepared using a freezing microtome. Brain sections from each group were subjected to immunofluorescence staining. Briefly, sections were permeabilized with 0.3% Triton X‐100, blocked with 5% bovine serum albumin for 2 h, and incubated overnight at 4°C with primary antibodies targeting GPX4 (affinity, DF6701, 1:400) and NeuN (proteintech, 66836‐1‐Ig, 1:200). The following day, sections were incubated with Alexa Fluor 488‐labeled goat anti‐rabbit IgG (Abcam, ab150077, 1:800) and Alexa Fluor 594‐labeled goat anti‐mouse IgG (Abcam, ab150116, 1:800) at room temperature for 1 h. Nuclei were stained with DAPI‐containing antiFade reagent, and sections were observed using a Nikon confocal microscope. Images were analyzed using NIKON viewer software.

### 2.9 Sample Preparation for LC–MS

For the untargeted metabolomics study, hippocampal tissues were extracted from sham group mice, KA group mice, and drug‐treated epileptic mice (*n* = 4 per group) on dry ice. The LC–MS sample preparation followed the protocol described by Yu et al. [31]. Approximately 10 mg of tissue was homogenized in 200 μL of water. To the 200 μL homogenate, 800 μL of a methanol and acetonitrile mixture (1:1 v/v) was added, vortexed for 30 s, and sonicated for 10 min. The samples were then kept at −20°C for 1 h, followed by centrifugation at 12,000 rpm for 15 min at 4°C. The supernatant was collected, and approximately 500 μL was transferred to a new tube and dried in a vacuum freeze dryer. The residue was reconstituted in 100 μL of a 1:1 acetonitrile and water mixture. The solution was vortexed in pre‐chilled water for 30 s and sonicated at 40 kHz for 5 min. After centrifugation at 12,000 rpm for 15 min at 4°C, 100 μL of the supernatant was transferred to a sample vial for LC–MS/MS analysis.

### 2.10 Metabolomic Analysis

Metabolite relative quantification was performed using a SHIMADZU CBM‐30A Lite LC system (Shimadzu Corporation, Kyoto, Japan) equipped with a Phenomenex Kinetex 2.6 μm C18 100A column (2.1 × 100 mm) coupled with a Q‐Exactive mass spectrometer (Thermo Fisher Scientific). The mobile phases were as follows: (A) a mixture of water: acetonitrile: methanol (3:1:1 v/v/v) with 5 mM ammonium acetate and (B) isopropanol, at a flow rate of 0.3 mL/min. The analysis used gradient elution with the following conditions: 0–0.5 min, 25% B; 0.5–1.5 min, 25%–40% B; 1.5–3 min, 40%–60% B; 3–13 min, 60%–98% B; 13–13.1 min, 98%–25% B; and 13.1–18 min, 25%–25% B. The column temperature was maintained at 40°C, and a sample volume of 2 μL was injected. The collision energy for fragmentation was set at 40 V.

Metabolite identification was performed using MSDIAL software (version 4.7), VIP values of metabolites were obtained using SIMCA software (version 14.1), and visualizations such as volcano plots and Venn diagrams were created using R software (version 4.3.1).

### 2.11 Molecular Docking

Molecular docking is a technique used to predict the preferred binding orientation of a ligand molecule to another molecule to form a stable complex. In this study, we employed semi‐flexible docking to form stable complexes, which is critical for elucidating mechanisms of action or screening lead compounds, making it a fundamental method in structure‐based drug design. AutoDock Vina 1.1.2 software was used to dock AST (PubChem CID: 5281224) with the protein NRF2 (Uniprot ID: Q60795). The 3D model of the protein was downloaded from the RCSB Protein Data Bank (http://www.rcsb.org/pdb). Preprocessing of the protein (removal of water molecules and excess ligands, addition of hydrogen atoms) was performed using PyMol 2.4. AutoDock Tools 1.5.6 was used to generate PDBQT files for docking simulations. The docking box size for NRF2 was set to 112 Å × 106 Å × 126 Å with a grid spacing of 1.00 Å, and the coordinates were set to *x*: 1.514, *y*: 4.293, *z*: −9.302. Other parameters were kept at default settings. The docking results were set to output the top 10 binding modes. The binding configuration with the lowest binding energy and highest clustering frequency was considered the most probable binding mode between the ligand and protein. Visualization of the docking results was performed using PyMol 2.4 to analyze the stability and interactions of the complexes.

### 2.12 Statistical Analysis

All data are presented as mean ± standard deviation (SD). The Shapiro–Wilk test was used to verify the normality of the data distribution. For normally distributed data, comparisons were made using unpaired *t*‐tests or one‐way analysis of variance (ANOVA). For non‐normally distributed data, comparisons were made using the Mann–Whitney *U* test or Kruskal–Wallis test. All analyses were conducted using GraphPad Prism 8.0 or R software, with *p* < 0.05 considered statistically significant.

## 3 Results

### 3.1 Involvement of Ferroptosis in the Development of Epilepsy

We modeled KA‐induced temporal lobe epilepsy (TLE) in mice at 12 h, 1 day, 3 days, and 7 days, and performed RT‐qPCR on hippocampal tissue. As shown in Figure 1C, the key markers of ferroptosis, PTGS2 and ACSL4, exhibited a significant increase at the mRNA level following KA induction, peaking between the first and third days. Additionally, another ferroptosis marker, GPX4, was significantly downregulated on the first and third days following KA‐induced epilepsy. This suggests that ferroptosis may play a role in the acute phase of TLE in mice, highlighting it as a potential therapeutic target for epilepsy. Based on these findings, we selected the third day post‐KA induction for subsequent experiments and designed the experimental workflow shown in Figure 1D. The primers used in these experiments are listed in Table S2.

### 3.2 AST Alleviates Neuronal Loss in KA‐Induced Epileptic Mice

**FIGURE 2:**
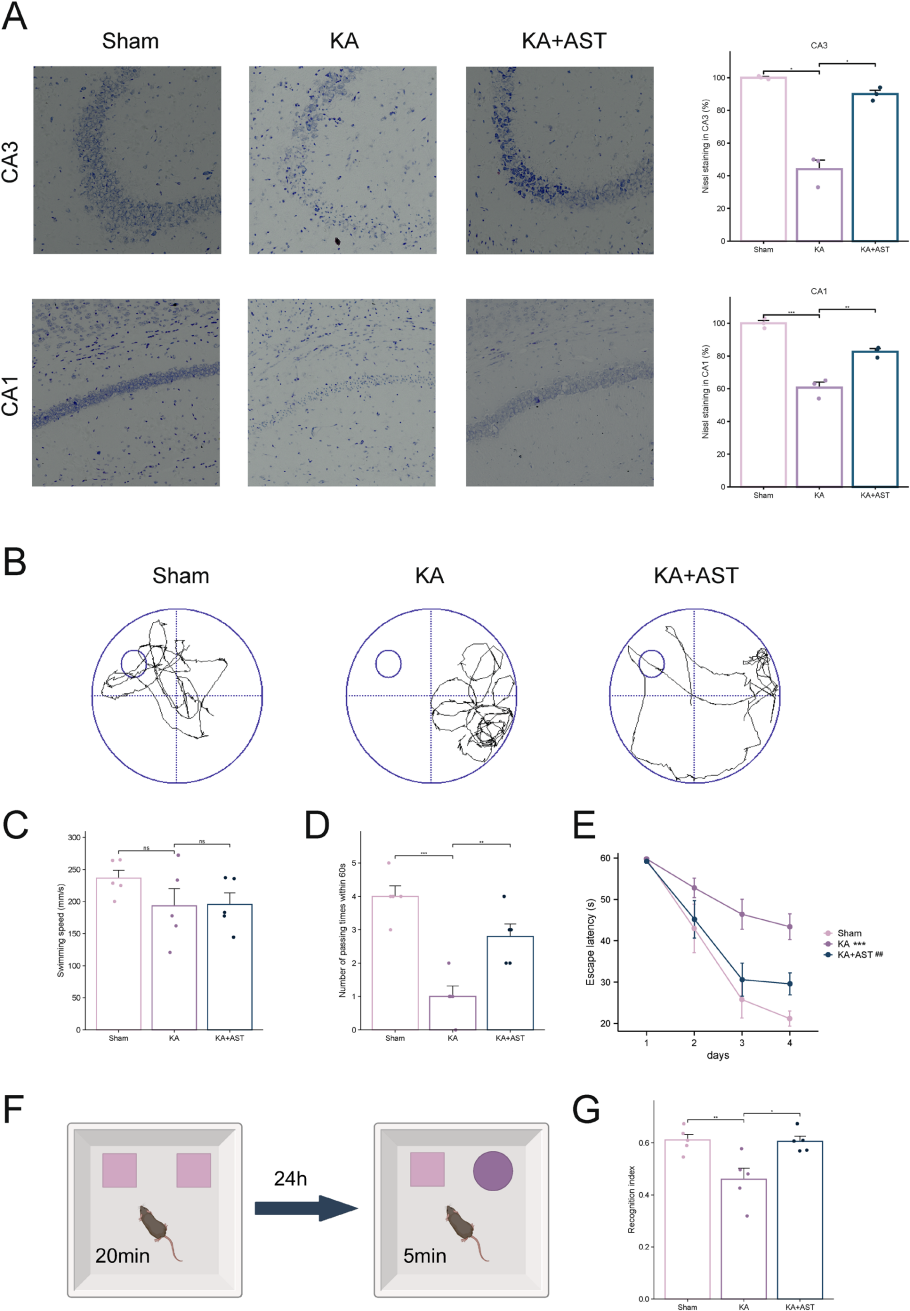
AST administration ameliorates neuronal loss and cognitive impairment in KA‐induced epileptic mice. (A) Representative Nissl staining images of hippocampal tissues from the three groups of mice (bar = 10 μm) with statistical data (*n* = 3). (B) Representative track plots on the fifth day of the Morris water maze test for the three groups of mice. (C) Swimming speed of the three groups of mice. (D) Platform crossings on the fifth day of the Morris water maze test for the three groups of mice. (E) Escape latency during training for the three groups of mice (*n* = 5). * indicates comparison between sham and KA groups, # indicates comparison between KA and KA + AST groups. (F) Schematic of the novel object recognition test. (G) Recognition index in the novel object recognition test for the three groups of mice (*n* = 5). Data are presented as mean ± SD. *p < 0.05, **p < 0.01, ***p < 0.001.

Nissl staining results (Figure 2A) revealed a significant trend of neuronal loss and deformation, with central nissl body dissolution in the CA3 and CA1 regions of the hippocampus in the KA group. In contrast, the KA + AST group demonstrated a reversal of neuronal loss and deformation in these hippocampal regions, with a greater number of intact surviving neurons. This indicates that AST possesses potential neuroprotective properties.

### 3.3 AST Reduces Cognitive Impairment in KA‐Induced Epileptic Mice

We utilized the MWM and novel object recognition tests to investigate the potential role of AST in mitigating learning and memory deficits during the acute phase of KA‐induced seizures in mice. As shown in Figure 2B–E, prior to the adaptation test, mice exhibited normal motor function with no significant differences in average swimming speed. The MWM navigation test revealed that the escape latency decreased from day 2 onwards across all groups, indicating some degree of learning and memory capability. However, differences in learning and memory abilities were observed between groups. Compared to the sham group, the KA group exhibited significantly prolonged escape latencies on day 4 (*p* < 0.001). Conversely, the AST‐treated group showed a significantly shorter escape latency compared to the KA group (*p* < 0.01). In the platform crossing test on day 5, the KA group showed a significant reduction in platform crossings compared to the sham group (*p* < 0.001), whereas the AST‐treated group exhibited a significant increase in platform crossings compared to the KA group (*p* < 0.05). Overall, AST treatment ameliorated cognitive deficits in KA‐induced epileptic mice.

The novel object recognition test workflow is shown in Figure 2F, and the results are depicted in Figure 2G. In this test, the KA group exhibited a significant reduction in the RI compared to the sham group (*p* < 0.05), indicating impaired exploratory ability toward novel objects. The AST‐treated group showed a significant increase in RI compared to the KA group (*p* < 0.05), suggesting that AST effectively mitigates learning and memory deficits induced by KA.

### 3.4 AST Mitigates Lipid Peroxidation and Mitochondrial Morphological Changes in KA‐Induced Epileptic Mice

**FIGURE 3:**
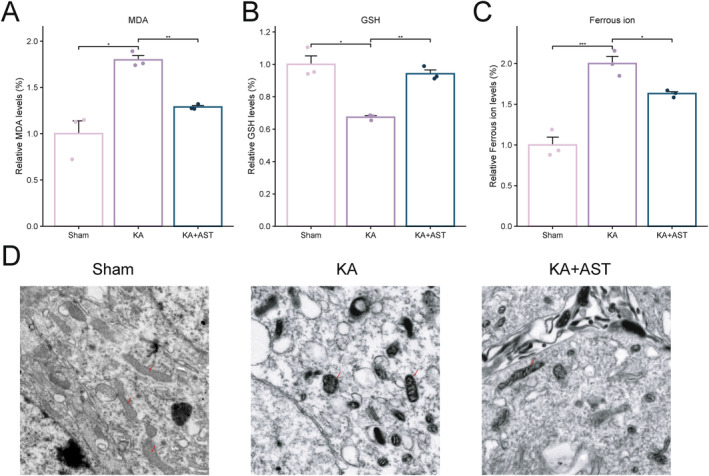
AST administration mitigates lipid peroxidation and mitochondrial abnormalities in KA‐induced epileptic mice. (A–C) Changes in MDA, GSH, and Ferrous ion levels in hippocampal tissues of the three groups of mice (*n* = 3). (D) Representative images of mitochondrial morphological changes in hippocampal tissues from the three groups of mice (bar = 0.5 μm, red arrows indicate representative mitochondria). Data are presented as mean ± SD. *p < 0.05, **p < 0.01, ***p < 0.001.

Lipid peroxidation and mitochondrial morphological changes are key characteristics of ferroptosis. To elucidate the anti‐lipid peroxidation effects of AST, we measured the levels of GSH, MDA, and ferrous ion in the hippocampal tissues using commercial kits. As shown in Figure 3A–C, the KA group exhibited a significant increase in MDA (*p* < 0.01) and ferrous ion levels (*p* < 0.001), along with a decrease in GSH levels (*p* < 0.05) compared to the sham group. These results indicate significant lipid peroxidation and ferrous ion accumulation in the hippocampus of KA‐induced epileptic mice, potentially contributing to ferroptosis. In contrast, AST treatment significantly reduced MDA and ferrous ion levels (*p* < 0.05) and increased GSH levels (*p* < 0.05) compared to the KA group, suggesting that AST reverses abnormal ferrous ion levels and lipid peroxidation induced by KA.

Figure 3D shows the transmission electron microscopy results. The sham group displayed intact hippocampal neuron structures with clear nuclear membranes, distinct nucleoli, and abundant, intact cytoplasmic organelles, including mitochondria. In contrast, the KA group exhibited irregular neuronal shapes, fragmented nuclear membranes, dispersed chromatin, degenerated and swollen mitochondria with rough outer membranes, blurred or absent cristae, and numerous vacuoles. Conversely, the KA + AST group displayed intact neuronal structures with largely intact nuclear membranes, occasional chromatin condensation, mildly swollen mitochondria with smooth outer membranes, and visible cristae, indicating that AST can reverse mitochondrial morphological changes induced by KA.

### 3.5 AST Inhibits Ferroptosis in KA‐Induced Epileptic Mice via the Nrf2/GPX4 Pathway

**FIGURE 4:**
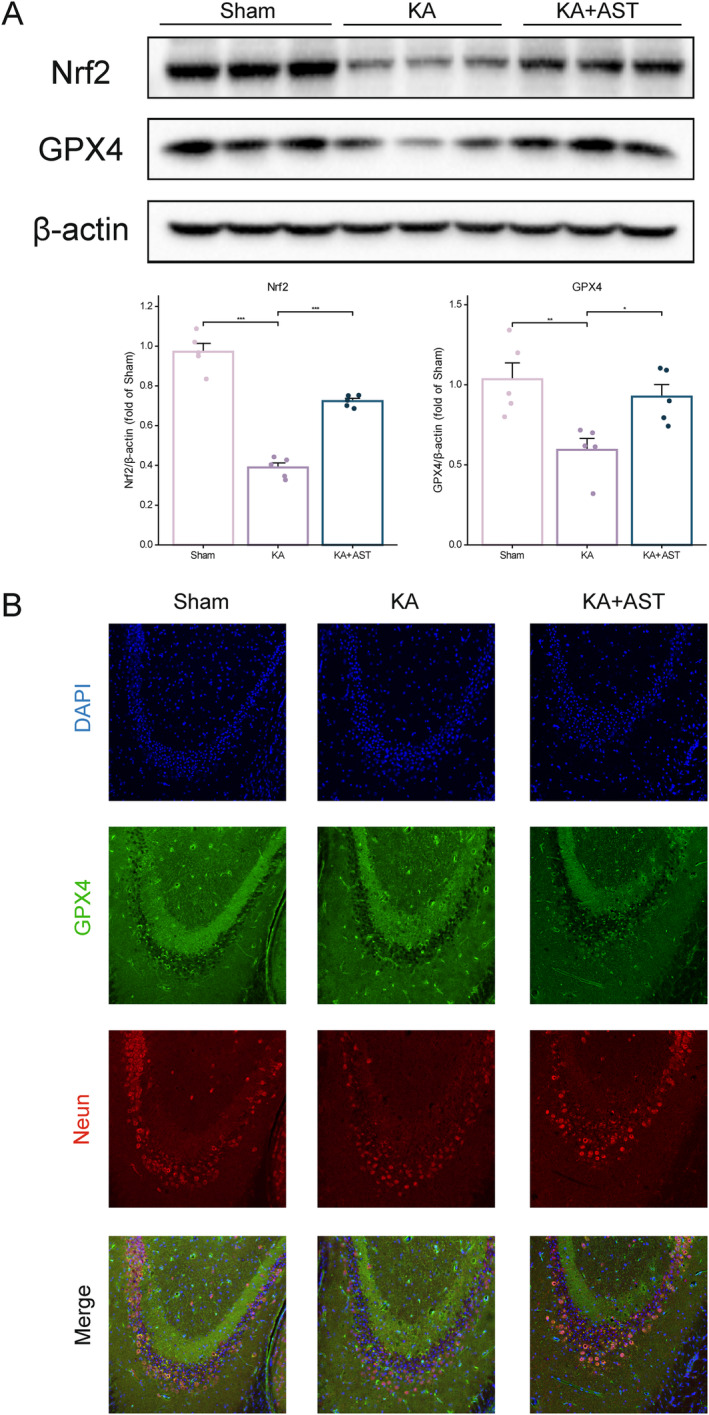
AST administration inhibits Nrf2‐mediated ferroptosis. (A) Representative Western blot images and statistical data of Nrf2 and GPX4 protein expression in hippocampal tissues of the three groups of mice (*n* = 5). (B) Representative immunofluorescence images showing GPX4 levels in the CA3 region of the hippocampus in the three groups of mice (bar = 100 μm). Data are presented as mean ± SD. *p < 0.05, **p < 0.01, ***p < 0.001.

We assessed the changes in Nrf2 and GPX4 protein levels through Western blotting. As shown in Figure 4A, the KA group exhibited significantly lower levels of Nrf2 and GPX4 in hippocampal tissues compared to the sham group (*p* < 0.01). However, AST pretreatment significantly reversed these changes (*p* < 0.05). Immunofluorescence staining further confirmed the changes in GPX4 levels (Figure 4B). Compared to the control group, the KA group showed a significant reduction in GPX4 levels in the CA3 region of the hippocampus, whereas AST pretreatment reversed this reduction. Additionally, NeuN staining revealed that GPX4 is expressed in neurons, and the number of NeuN‐expressing cells decreased under KA induction, which was rescued by AST pretreatment. These findings strongly suggest that AST inhibits KA‐induced ferroptosis through the Nrf2/GPX4 pathway.

### 3.6 Untargeted Metabolomics

**FIGURE 5:**
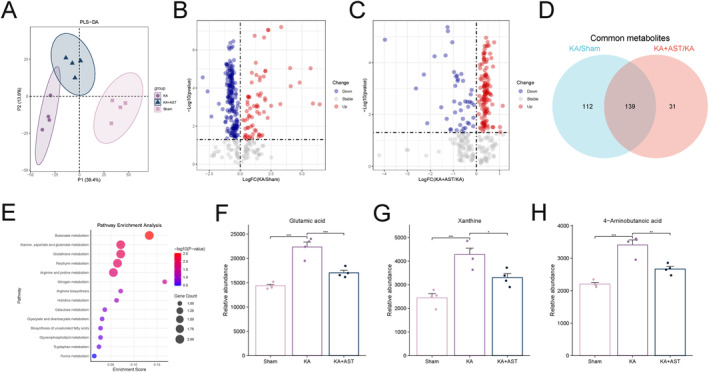
Untargeted lipidomics analysis. (A) PLS‐DA plot. (B) Volcano plot of differentially expressed metabolites between the sham and KA groups. (C) Volcano plot of differentially expressed metabolites between the KA and KA + AST groups. (D) Venn diagram showing 139 common differentially expressed metabolites. (E) KEGG pathway enrichment bubble plot for identified metabolites among the 139 differentially expressed metabolites. (F–H) Changes in the relative abundance of Glutamic acid, Xanthine, and 4‐Aminobutanoic acid in hippocampal tissues of the three groups of mice (*n* = 4). Data are presented as mean ± SD. *p < 0.05, **p < 0.01, ***p < 0.001.

Untargeted metabolomics was employed to analyze metabolic changes in the hippocampal tissues of KA‐induced epileptic mice. The PLS‐DA plot revealed significant group differences among the three mouse groups (Figure 5A). Metabolites with VIP > 1 were included in subsequent analyses. The volcano plot demonstrated 251 differentially expressed metabolites between the KA and sham groups, with 181 downregulated and 70 upregulated (Figure 5B). Similarly, 170 differentially expressed metabolites were identified between the KA and AST‐treated groups, with 44 downregulated and 126 upregulated (Figure 5C). A Venn diagram identified 139 common differentially expressed metabolites, suggesting that these metabolites were aberrant in the KA group and reversed by AST treatment (Figure 5D). Among these, 39 metabolites were annotated. KEGG pathway enrichment analysis indicated significant activation of butanoate metabolism and glutathione metabolism pathways (Figure 5E). Further exploration of these pathways revealed that ferroptosis‐related metabolites, such as xanthine, 4‐aminobutanoic acid, and glutamic acid, were significantly elevated in the KA group, whereas AST pretreatment reduced their relative abundance (Figure 5F–H). Given that the glutathione metabolism pathway is a key pathway in ferroptosis and is related to the GPX4 pathway, these findings confirm that AST can mitigate the metabolic changes induced by KA in epileptic mice, changes that may be associated with ferroptosis.

### 3.7 AST Reduces Ferroptosis in Hippocampal Neurons of KA‐Induced Epileptic Mice via the Nrf2/GPX4 Pathway

**FIGURE 6:**
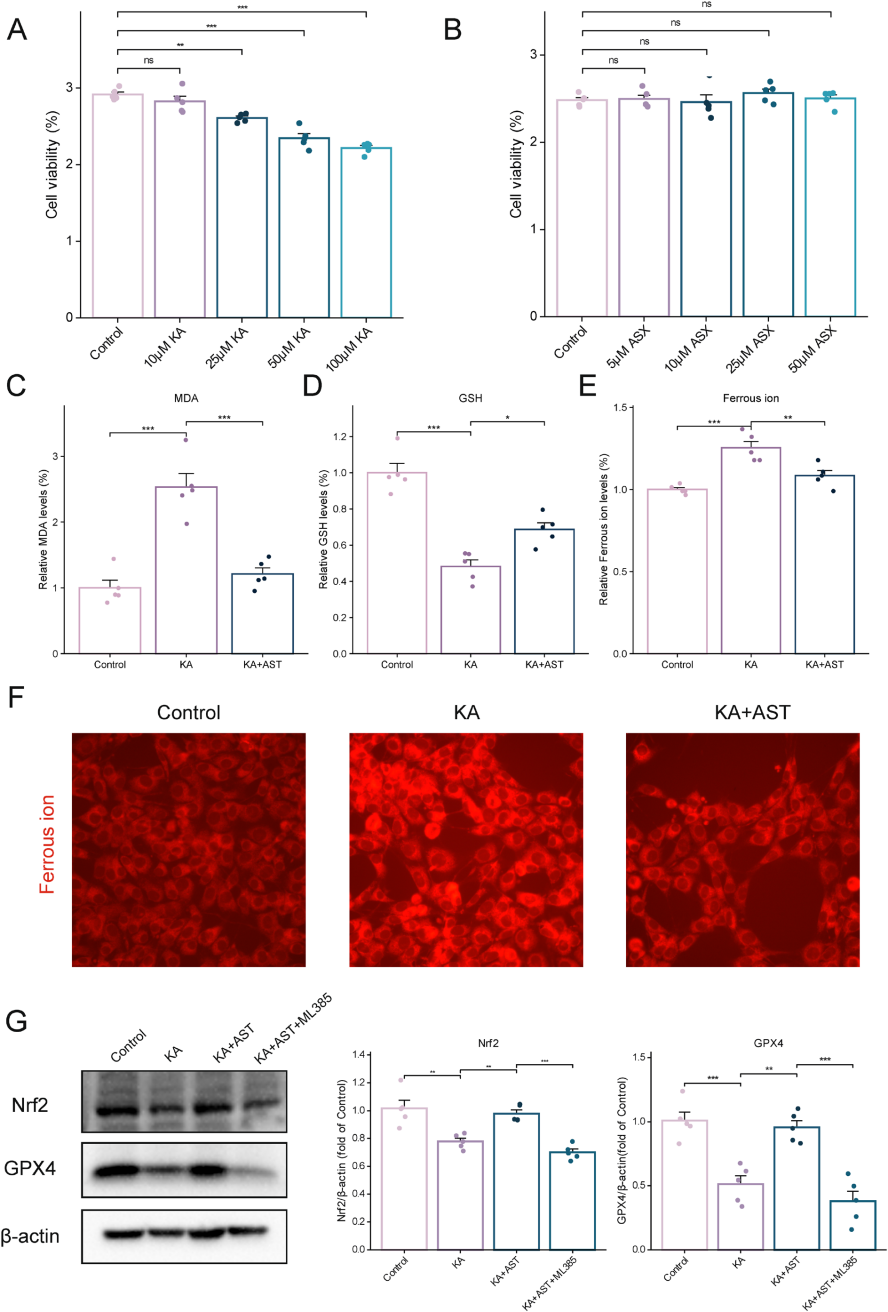
AST administration provides neuroprotection against KA‐induced HT22 cell damage by inhibiting Nrf2‐mediated ferroptosis. (A) Effect of different concentrations of KA on HT22 cell viability. (B) Effect of different concentrations of AST on HT22 cell viability. (C–E) Changes in MDA, GSH, and Ferrous ion levels in HT22 cells of the three groups (*n* = 5). (F) Representative images of Ferrous ion fluorescence in HT22 cells of the three groups (bar = 50 μm). (G) Representative Western blot images and statistical data of Nrf2 and GPX4 protein expression in HT22 cells of the three groups (*n* = 5). Data are presented as mean ± SD. *p < 0.05, **p < 0.01, ***p < 0.001.

To model neuronal epilepsy, HT22 cells were induced with KA as described in previous studies. We established respective groups, and CCK8 assay results showed a dose‐dependent decrease in cell viability in the KA group compared to the control group (Figure 6A). Various concentrations of AST did not affect HT22 cell viability (Figure 6B). Consequently, a 50 μM KA concentration was chosen for subsequent experiments. Using commercial assay kits, we measured ferrous ion, MDA, and GSH levels in the KA‐induced neuronal death model, further verifying lipid peroxidation changes. The results showed a significant increase in ferrous ion and MDA levels and a significant decrease in GSH levels in the KA group compared to the control group (*p* < 0.001). AST pretreatment significantly reversed these changes (*p* < 0.05), as shown in Figure 5C–E. To further elucidate ferrous ion changes, a fluorescent assay was used, indicating higher fluorescence signals in the KA group, which were reduced by AST pretreatment (Figure 5F). Western blotting confirmed our hypothesis, showing reduced Nrf2 and GPX4 expression in KA‐treated HT22 cells compared to controls (*p* < 0.001). However, AST pretreatment significantly reversed these changes (*p* < 0.01). Using an Nrf2 inhibitor (ML385, 5 μM), we found that the neuroprotective effect of AST was attenuated upon Nrf2 inhibition (*p* < 0.001), suggesting that AST exerts neuroprotective effects in KA‐treated HT22 cells via Nrf2 (Figure 6G). Overall, these findings, consistent with in vivo results, strongly indicate that AST confers neuroprotection by inhibiting Nrf2‐mediated ferroptosis.

### 3.8 AST Inhibits Ferroptosis of Hippocampal Neurons in KA‐Induced Epileptic Female Mice by Activating the Nrf2/GPX4 Signaling Pathway

To further ensure the rigor of the research, we also conducted experiments on female mice. Female mice of the same age were selected, grouped, and tested according to the aforementioned experimental protocol. The results showed that AST administration not only delayed cognitive impairment and hippocampal neuron loss in female epileptic mice but also reduced ferroptosis indicators such as lipid peroxidation, iron accumulation, and mitochondrial morphological changes. Additionally, Western blot experiments indicated that this pharmacological effect might be achieved through the activation of the Nrf2/GPX4 signaling pathway. In summary, AST can also inhibit ferroptosis in hippocampal neurons of female epileptic mice by activating the Nrf2/GPX4 signaling pathway, as shown in Figure S1.

### 3.9 Bioinformatics Analysis Reveals That AST Mitigates Epilepsy via Nrf2‐Mediated Ferroptosis

The Comparative Toxicogenomics Database (CTD) is a public database aimed at enhancing the understanding of how environmental chemicals affect human health [32]. It integrates data on chemical, gene, and disease interactions, providing a comprehensive information platform to advance research in toxicology, genomics, and bioinformatics. From the CTD database, we extracted 126 genes associated with AST, which are believed to be pivotal in mediating its effects on the human body.

The GeneCards database is a comprehensive bioinformatics resource offering detailed information on all known and predicted disease‐ or phenotype‐related genes [33]. We retrieved 6184 epilepsy‐related protein‐coding targets from GeneCards (https://www.genecards.org) using “Epilepsy” as the keyword. After filtering targets based on relevance scores, 3090 targets were selected.

FerrDB is a database focused on iron‐related genes and proteins, providing extensive information on iron metabolism‐associated diseases, proteins, genes, pathological processes, and ferroptosis‐related molecular mechanisms [34]. The latest version, FerrDB V2, lists 621 ferroptosis‐related genes, including 264 drivers, 238 suppressors, 9 biomarkers, and 110 undefined genes.

**FIGURE 7:**
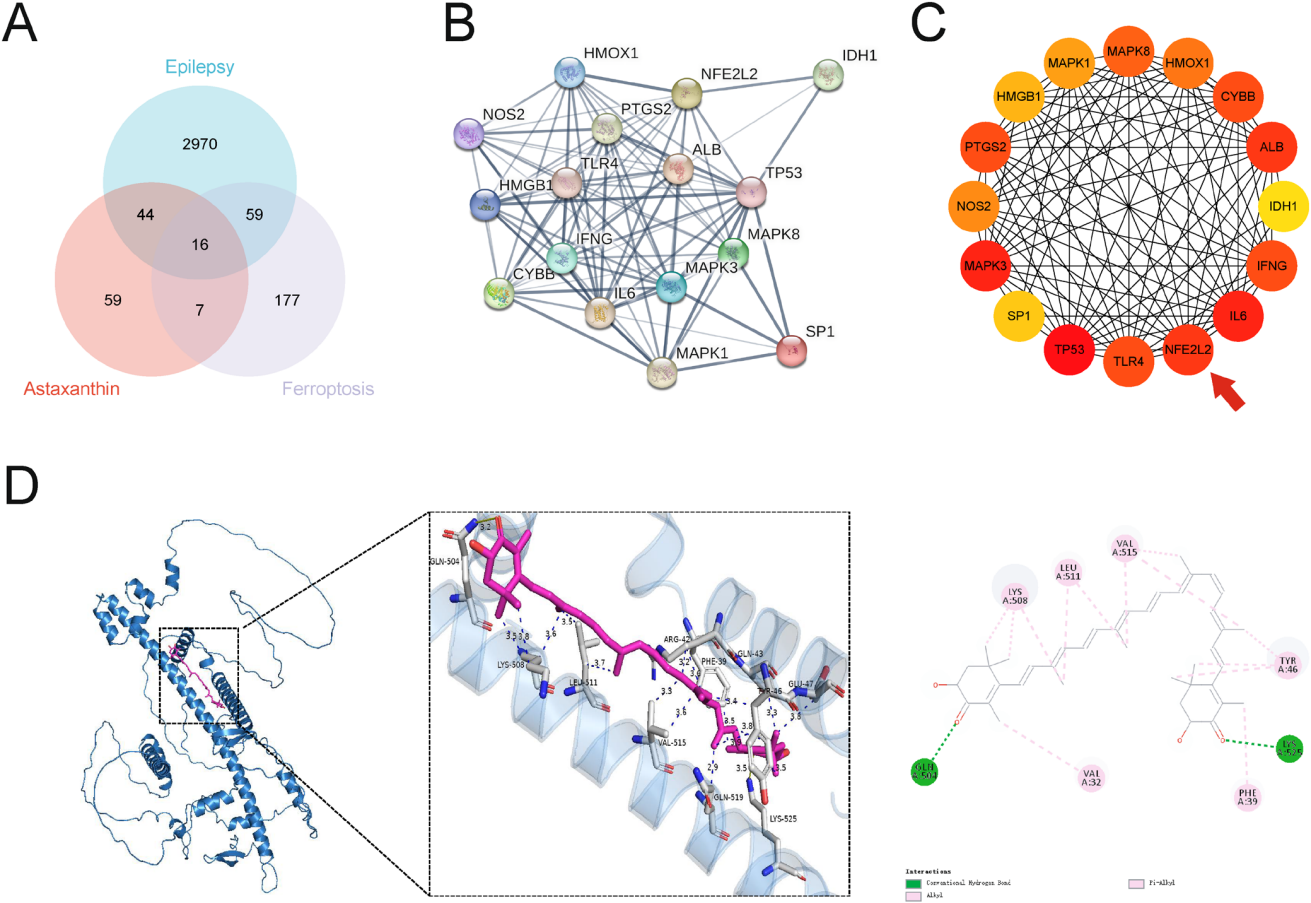
Nrf2‐mediated ferroptosis may be involved in the pathogenesis of epilepsy. (A) Venn diagram showing common genes derived from CTD, FerrDB, and GeneCards databases. (B) PPI network visualization of the 16 common genes. (C) Key importance of the 16 genes generated by the cytoHubba plugin in Cytoscape, with deeper red indicating higher importance. (D) 3D and 2D molecular docking images showing the interaction sites between AST and Nrf2 protein.

Through intersection analysis of genes from these three databases, we identified 16 common genes (Figure 7A). Subsequent protein interaction network construction highlighted the key role of the NFE2L2 gene in the subnetwork (Figure 7B,C), suggesting that Nrf2‐mediated ferroptosis likely participates in epilepsy development.

Additionally, molecular docking was employed to explore the optimal binding mode between AST and Nrf2 (Figure 7D). Docking results provided details on binding energy, interaction forces, and bond lengths (Table S3). AST exhibited a binding energy of −9.6 Kcal/mol with Nrf2. Most of the free hydroxyl groups in the AST structure interacted with the active site of the protein. The 2D interaction analysis revealed that AST's hydrophobic carbon chain formed Van der Waals interactions with nearby amino acids. The Nrf2 active site comprises amino acid residues such as Phe, Arg, Gln, Tyr, Glu, Lys, Leu, and Val. The binding pocket contains hydrophobic residues Phe, Leu, and Val. There were 17 hydrophobic interactions in the binding pocket, indicating significant contributions to binding stability. Notably, AST interacted with aromatic residues Phe and Tyr. These molecular docking results underscore the potential of AST in modulating key functions of Nrf2, providing strong motivation for its application development.

## 4 Discussion

Epilepsy is a common neurological disorder with various subtypes. This study specifically focused on TLE, the most prevalent form of focal epilepsy, characterized by recurrent, unprovoked seizures originating in the temporal lobe, often involving the hippocampus [35]. The pathogenesis of TLE involves complex signaling networks and various effector molecules. In foundational research, commonly used drugs to induce epilepsy in animal models include lithium chloride, pilocarpine, KA, and pentylenetetrazol. These chemicals generate epileptiform activity by affecting brain regions in mice, thereby creating epilepsy models. In this study, we used the widely recognized KA‐induced mouse model to simulate TLE for exploratory research.

AST was approved as a food additive by the US Food and Drug Administration (FDA) in 1999 [36]. Several studies in the field of neuroscience have shown that AST can alleviate oxidative stress and reduce neuroinflammatory responses through the activation of the Nrf2 pathway [37, 38, 39, 40]. Recent research indicates that AST rescues memory impairments in rats with vascular dementia by protecting against neuronal death in the hippocampus [41], and it can also alleviate cognitive deficits in Alzheimer's disease models [42] and dopaminergic neuronal damage in Parkinson's disease models [23]. This suggests that AST may serve as a neuroprotective agent in neurodegenerative diseases [43]. Factors such as inflammatory response and oxidative stress are reported to exacerbate neuronal death in TLE. Although AST has been shown to benefit rat epilepsy models induced by amygdala kindling under oxidative stress conditions [44], and to prevent neuronal excitotoxicity by inhibiting glutamate receptors and improving mitochondrial function [45], there is a dearth of in‐depth studies on the mechanisms of AST in TLE.

In recent years, ferroptosis has been widely reported to be associated with various neurological disorders, including stroke [46], Alzheimer's disease [47], and Parkinson's disease [48]. Additionally, ferroptosis has been detected in epilepsy models. Consistent with our findings, we observed abnormal expression of ferroptosis‐related proteins ACSL4, PTGS2, and GPX4 in the hippocampal tissues of KA‐induced epileptic mice, indicating the involvement of ferroptosis in epilepsy. The Nrf2/GPX4 signaling pathway is currently recognized as a crucial regulator of ferroptosis [49, 50]. Studies suggest that targeting the Nrf2/GPX4 pathway to inhibit ferroptosis could be a promising therapeutic strategy. Thus, we explored whether AST could inhibit ferroptosis via the Nrf2/GPX4 pathway to have a neuroprotective effect on epilepsy.

At the animal level, lipid peroxidation, mitochondrial morphological changes, and alterations in ferroptosis‐related proteins are hallmark indicators of ferroptosis. We investigated these through various experiments including PCR, Western blot, electron microscopy, and assays. The results demonstrated the presence of ferroptosis in the hippocampal tissues of KA‐induced mice, potentially mediated by Nrf2. Treatment with AST showed neuroprotective effects and improved cognitive function in KA‐induced epileptic mice, likely by inhibiting Nrf2‐mediated ferroptosis. Bioinformatics analysis corroborated our results, indicating that AST might act on Nrf2‐mediated ferroptosis in epilepsy. Additionally, untargeted metabolomics revealed that the glutathione metabolism pathway is involved in AST's neuroprotective role. GPX4, a monomeric glutathione peroxidase, plays a crucial role in ferroptosis by reducing peroxides in complex lipids [51, 52]. Untargeted metabolomics indicated that metabolites such as xanthine, 4‐aminobutanoic acid, and glutamic acid, which were significantly altered in the epilepsy model, were reversed by AST treatment. Xanthine, a purine degradation intermediate, is considered a central nervous system activator. Administration of xanthine has been reported to induce hippocampal‐dependent spatial memory deficits and anxiety‐like behaviors in mice [53]. Reactive oxygen species (ROS), associated with ferroptosis, can be produced by xanthine oxidase [54]. Previous reports have shown that metabolic pathways involving glutathione and purine metabolism contribute to ferroptosis in TM4 cells exposed to PM2.5 [55]. 4‐Aminobutanoic acid, also known as gamma‐aminobutyric acid, is an inhibitory neurotransmitter that maintains inhibitory tone to counterbalance neuronal excitability. Disruption of this balance can lead to seizures [56]. Although some studies show that GABA levels significantly decrease in the hippocampus during seizures [57, 58, 59], others report increases in glutamate, aspartate, and GABA during epilepsy [60], suggesting that GABA changes may vary across different stages of the disease, warranting further investigation. Glutamate, known for its excitotoxicity in epilepsy, has been reported to be upregulated in various epilepsy models [61, 62, 63]. Our results indicate that AST mitigates KA‐induced neuronal damage, possibly by reducing increased glutamate levels in the hippocampus. Interestingly, glutamate is often used to model excitotoxicity in HT22 cells, which is related to ferroptosis in several studies [64, 65], consistent with our findings. This further supports the hypothesis that AST inhibits neuronal death in epilepsy by activating the Nrf2/GPX4 signaling pathway to inhibit ferroptosis.

At the cellular level, we first investigated the toxicity of AST on HT22 neurons, confirming that AST at various concentrations is nontoxic to HT22 cells. Niu et al. [66] reported poor cell permeability of AST, which we also observed as red AST crystals at the bottom of the cell culture plate, suggesting that the actual amount penetrating the cells may be much lower than the applied dose. Simultaneously, we stimulated HT22 neuronal cells with varying concentrations of KA, and the results demonstrated that cell viability decreased as the KA concentration increased. The CCK‐8 assay, ferroptosis‐related kits, and Western blot experiments all indirectly supported that this neuronal death could be induced by ferroptosis, and that administration of AST could reverse this neuronal death. To verify that Nrf2 activation mediates AST's neuroprotective effects on HT22 cells, we inhibited Nrf2 expression using an inhibitor. As noted, inhibiting Nrf2 blocked KA‐induced neurotoxicity and AST‐mediated rebound expression of ferroptosis‐related proteins, suggesting that Nrf2 activity mediates AST's neuroprotective effects. Additionally, the protective effects of AST on neurons were reversed when Nrf2 was inhibited.

However, our study has some limitations and unresolved questions. Firstly, we only used Nrf2 inhibitors in cell experiments and did not block Nrf2 in animal experiments, lacking this part of the study. Consequently, the relationship between AST‐mediated improvement in epilepsy through Nrf2‐mediated ferroptosis remains speculative, and other pathways may also be involved, warranting further research. Secondly, clinical trials are needed to confirm the therapeutic effects of AST in epilepsy patients. Lastly, due to database limitations, many metabolites in the untargeted metabolomics results remain unidentified, and the enrichment results are merely suggestive.

## 5 Conclusion

Overall, our in vitro and in vivo results indicate that AST exerts neuroprotective effects on KA‐induced epileptic mice and HT22 cells, likely by inhibiting ferroptosis. Specifically, AST activates the Nrf2/GPX4 signaling pathway to regulate glutathione metabolism and inhibit ferroptosis, offering a novel therapeutic strategy for targeting epilepsy.

## Conflicts of Interest

The authors declare no conflicts of interest.

## Supporting information


**Figure S1.** AST inhibits ferroptosis of hippocampal neurons in KA‐induced epileptic female mice by activating the Nrf2/GPX4 signaling pathway. (A, B) Representative Western blot images and statistical data of Nrf2 and GPX4 protein expression in hippocampal tissues of the three groups of female mice (*n* = 3). (C) Representative track plots on the fifth day of the Morris water maze test for the three groups of mice. (D) Escape latency during training for the three groups of mice. * indicates comparison between sham and KA groups, # indicates comparison between KA and KA + AST groups. (E) Platform crossings on the fifth day of the Morris water maze test for the three groups of mice. (F) Recognition index in the novel object recognition test for the three groups of mice. (G–I) Changes in Ferrous ion, MDA and GSH levels in hippocampal tissues of the three groups of mice (*n* = 3). (J, K) Representative Nissl staining images of hippocampal tissues from the three groups of mice (bar = 10 μm) with statistical data. (L) Representative images of mitochondrial morphological changes in hippocampal tissues from the three groups of mice (bar = 0.2 μm). Data are presented as mean ± SD. *p < 0.05, **p < 0.01, ***p < 0.001.

**Figure S2.** Original and uncut images of Western blot.

**Table S1.** The detailed information of the antibodies used for Western blot.

**Table S2.** The detailed information of the primer used for RT‐qPCR.

**Table S3.** The detailed information on the molecular docking experiment of astaxanthin and Nrf2.

## Data Availability

The data that support the findings of this study are available from the corresponding author upon reasonable request.
