# Supplementary figures and images for: Astaxanthin Inhibits Ferroptosis of Hippocampal Neurons in Kainic Acid‐Induced Epileptic Mice by Activating the Nrf2/GPX4 Signaling Pathway
Source: CNS Neurosci Ther. 2025 Feb 17;31(2):e70238. doi: 10.1111/cns.70238 (PMC11831069; doi:10.1111/cns.70238)

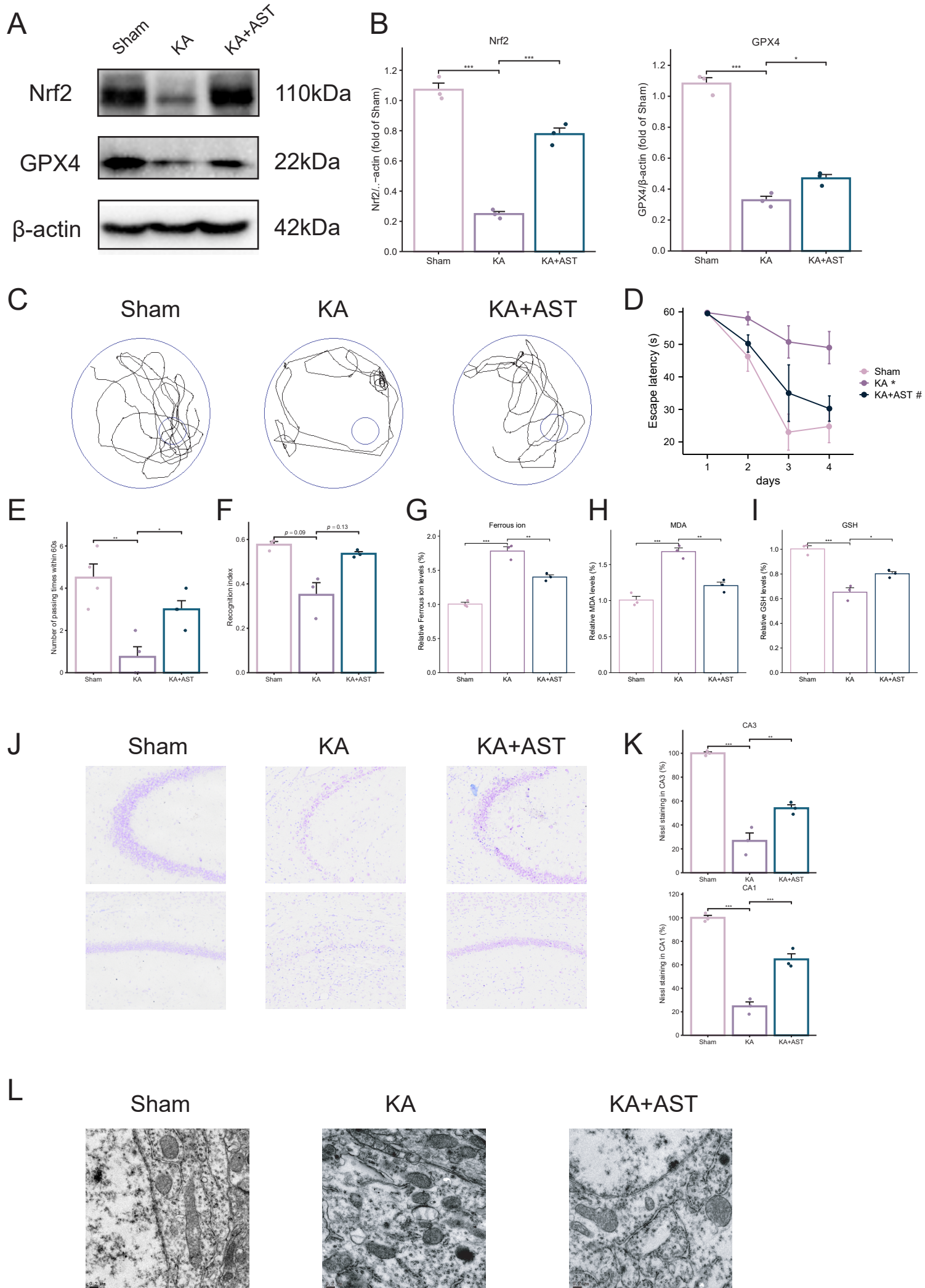

Supplement: Supplementary file 1 — Figure S1. AST inhibits ferroptosis of hippocampal neurons in KA‐induced epileptic female mice by activating the Nrf2/GPX4 signaling pathway. (A, B) Representative Western blot images and statistical data of Nrf2 and GPX4 protein expression in hippocampal tissues of the three groups of female mice (n = 3). (C) Representative track plots on the fifth day of the Morris water maze test for the three groups of mice. (D) Escape latency during training for the three groups of mice. * indicates comparison between sham and KA groups, # indicates comparison between KA and KA + AST groups. (E) Platform crossings on the fifth day of the Morris water maze test for the three groups of mice. (F) Recognition index in the novel object recognition test for the three groups of mice. (G–I) Changes in Ferrous ion, MDA and GSH levels in hippocampal tissues of the three groups of mice (n = 3). (J, K) Representative Nissl staining images of hippocampal tissues from the three groups of mice (bar = 10 μm) with statistical data. (L) Representative images of mitochondrial morphological changes in hippocampal tissues from the three groups of mice (bar = 0.2 μm). Data are presented as mean ± SD. *p < 0.05, **p < 0.01, ***p < 0.001. [file CNS-31-e70238-s002.pdf]

Figure 4 A

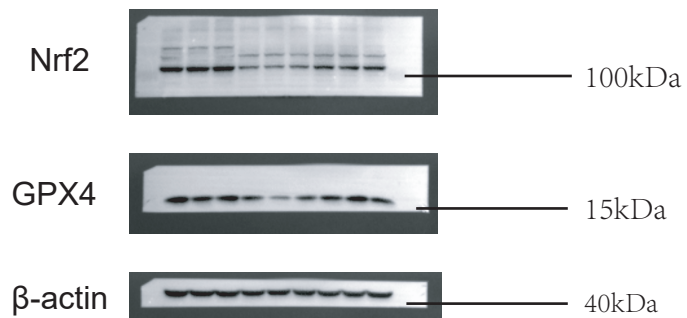

Figure 6 G

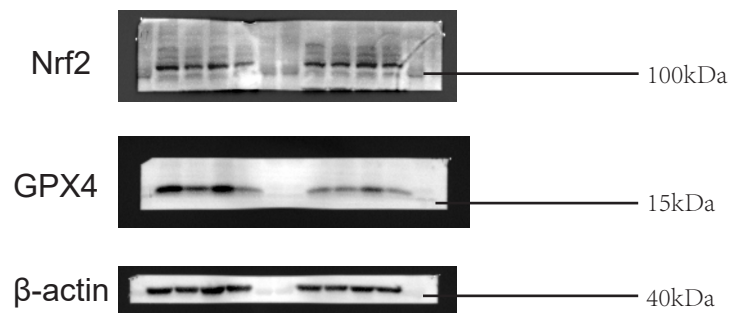

Supplemental Figure 1

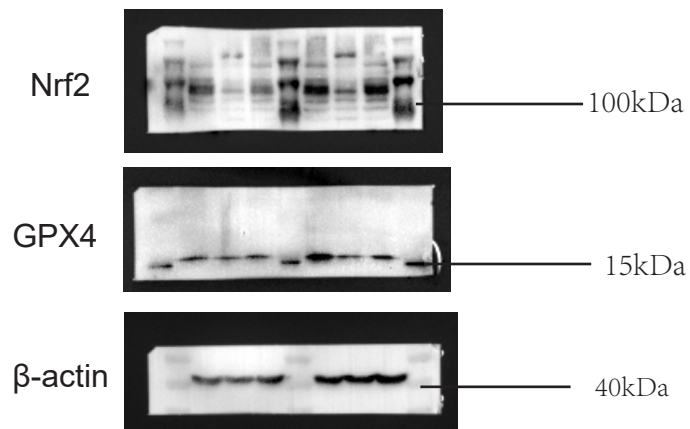

Supplement: Supplementary file 2 — Figure S2. Original and uncut images of Western blot. [file CNS-31-e70238-s001.pdf]
